# Early Effects of Bronchoscopic Cryotherapy in Metastatic Non-Small Cell Lung Cancer Patients Receiving Immunotherapy: A Single-Center Prospective Study

**DOI:** 10.3390/diagnostics15020201

**Published:** 2025-01-17

**Authors:** Gediminas Vasiliauskas, Evelina Žemaitė, Erika Skrodenienė, Lina Poškienė, Gertrūda Maziliauskienė, Aurimas Mačionis, Skaidrius Miliauskas, Donatas Vajauskas, Marius Žemaitis

**Affiliations:** 1 Department of Pulmonology, Lithuanian University of Health Sciences, 44307 Kaunas, Lithuania; skaidrius.miliauskas@lsmu.lt (S.M.); marius.zemaitis@lsmu.lt (M.Ž.); 2 Department of Laboratory Medicine, Lithuanian University of Health Sciences, 44307 Kaunas, Lithuania; evelina.zemaite@lsmu.lt (E.Ž.); erika.skrodeniene@lsmu.lt (E.S.); 3 Department of Pathology, Lithuanian University of Health Sciences, 44307 Kaunas, Lithuania; lina.poskiene@lsmu.lt; 4 Department of Radiology, Lithuanian University of Health Sciences, 44307 Kaunas, Lithuania; gertruda.maziliauskiene@lsmu.lt (G.M.); aurimas.macionis@lsmu.lt (A.M.); donatas.vajauskas@lsmu.lt (D.V.)

**Keywords:** lung cancer, cryotherapy, immunotherapy

## Abstract

**Background/Objectives:** Cryotherapy is used for local tissue destruction through rapid freeze–thaw cycles. It induces cancer cell necrosis followed by inflammation in the treated tumor microenvironment, and it stimulates systemic adaptive immunity. Combining cryotherapy with immunotherapy may provide a sustained immune response by preventing T cell exhaustion. **Methods:** Fifty-five patients with metastatic non-small cell lung cancer who had received no prior treatment were randomized into two groups in a 1:1 ratio: the bronchoscopic cryotherapy group or the control group. Patients received up to four cycles of pembrolizumab as monotherapy or in combination with platinum-based chemotherapy. Immune-related adverse events (irAEs), complications, tumor size changes, overall response rate (ORR), and disease control rate (DCR) were evaluated. **Results:** Lung tumors, treated with cryotherapy, demonstrated continuous reduction from the baseline (22.4 cm^2^ vs. 14.4 cm^2^ vs. 10.2 cm^2^, *p* < 0.001). Similar changes were observed in pulmonary tumors in the control group (19.0 cm^2^ vs. 10.0 cm^2^, *p* < 0.001). The median change in pulmonary tumors between two groups was not significant (−42.9% vs. −27.7%, *p* = 0.175). No significant differences were observed in the ORR (28.6% vs. 23.1%, *p* = 0.461) or target lesion decrease (−24.0% vs. −23.4%, *p* = 0.296) between the groups. However, the DCR was significantly higher in the cryotherapy group (95.2% vs. 73.1%, *p* = 0.049). No cases of serious bleeding during cryotherapy or pneumothorax were observed. Six patients (25.0%) in the cryotherapy group and eight (26.7%) in the control group experienced irAEs. **Conclusions:** Our study demonstrated that combined bronchoscopic cryotherapy and immunotherapy with or without chemotherapy may reduce the rate of progressive disease in metastatic non-small cell lung cancer patients while maintaining a satisfactory safety profile.

## 1. Introduction

Lung cancer remains the most frequently diagnosed form of cancer, with nearly 2.5 million new cases in 2022, and it is a leading cause of cancer-related mortality, responsible for an estimated 1.8 million deaths worldwide [1]. The overall survival rate for non-small cell lung cancer (NSCLC) remains low, largely due to late diagnosis, as 45% of patients present with stage IV disease [2]. Since systemic treatment remains the cornerstone in these cases, the continued development and refinement of targeted therapies and immunotherapies is imperative to improve patient survival.

The tumor immune microenvironment is a heterogeneous and dynamic landscape that influences cancer pathogenesis and response to therapy [3]. An inflamed or “hot” tumor microenvironment has been associated with better prognosis and response to immunotherapy compared to immunologically “cold” tumors [4]. These differences help explain the observed objective response rate of only about 45% in NSCLC patients treated with immunotherapy, also highlighting the need for further studies aimed at transforming “cold” tumors into “hot” ones [5,6,7].

Cryotherapy is used for local tissue destruction via rapid freeze–thaw cycles [8,9]. This process leads to the formation of intracellular ice crystals, which damage cell membranes and organelles, as well as freezing extracellular water, causing fluid shifts and cell dehydration [9,10]. As malignant cells die in this manner, their neoantigens remain in the body, acting as a form of vaccination. These neoantigens are absorbed by antigen-presenting cells, stimulating adaptive immunity. While this process may induce inflammation in the treated tumor microenvironment, creating a more favorable environment for an anti-tumor immune response, reports have also noted the shrinking of distant untreated metastases. However, subsequent research has shown that cryotherapy alone does not reliably create or sustain this effect [11].

Murine models have demonstrated that cryotherapy can cause the upregulation of programmed cell death protein 1 (PD-1) on T cells and programmed death-ligand 1 (PD-L1) on cancer cells, which ultimately inhibits the systemic anti-tumor response [12,13]. The addition of immune checkpoint inhibitors may counteract this mechanism and induce a stronger anti-tumor immune response. However, current clinical research investigating the synergistic mechanisms of cryotherapy and immunotherapy consists only of a few case reports and small studies [14,15,16,17].

The clinical benefits of combining cryotherapy with immune checkpoint inhibitor therapy remain underexplored. This study aims to evaluate the safety and effectiveness of this treatment combination for metastatic NSCLC, in search of more effective therapeutic options.

## 2. Materials and Methods

### 2.1. Study Design

Patients with metastatic non-small cell lung cancer and eligible for first-line systemic treatment including immunotherapy were enrolled. Consenting men and women who were at least 18 years old were eligible if they had histologically confirmed metastatic non-small cell lung cancer; no activating EGFR or ALK gene mutations; known PD-L1 expression on tumor cells; scored 0 to 1 according to Eastern Cooperative Oncology Group (ECOG) performance status score [18]; at least one pulmonary lesion, measurable according to the Response Evaluation Criteria in Solid Tumors, version 1.1 (RECIST 1.1) criteria [19] and reachable via flexible bronchoscopy; received no surgical treatment, radiotherapy, or chemotherapy for at least 12 months before the study and no prior immunotherapy. Patients with brain metastases were permitted to enroll after Gamma Knife radiosurgery.

Patients were excluded if they were unable or unwilling to undergo bronchoscopy, were previously diagnosed with autoimmune or immunosuppressive diseases, were currently treated with immunosuppressive drugs or systemic corticosteroids (with prednisolone equivalent doses exceding 10 mg daily), were positive for hepatitis B virus surface antigen or hepatitis C virus antibody, had active HIV, or had pulmonary tuberculosis infection.

Informed consent was obtained from all subjects involved in the study. Patients were randomized in a 1:1 ratio to receive bronchoscopic cryotherapy. As per standard care, patients further received pembrolizumab monotherapy (for PD-L1 tumor proportion score equal to or greater than 50%) or pembrolizumab and platinum-based chemotherapy (for PD-L1 tumor proportion score less than 50%). Patients who were assigned to the cryotherapy group were offered to continue as controls if the procedure was not technically successful.

This study was conducted in accordance to the guidelines of the Declaration of Helsinki and approved by the Kaunas Regional Biomedical Research Ethics Committee. This study was registered in the United States National Institute of Health trial registry (https://clinicaltrials.gov/) under identifier NCT06000358.

### 2.2. Procedure

The cryotherapy procedure was performed 7 (±1) days before the start of systemic treatment via a flexible bronchoscope (Olympus Corporation, Tokyo, Japan), under conscious sedation and visual (for endobronchial cryotherapy) or radial endobronchial ultrasound (EBUS) and fluoroscopy control (for transbronchial cryotherapy), ensuring the correct position of the cryoprobe in the tumor. After being placed in the correct position, the cryoprobe was cooled for at least 30 s, using an ERBECRYO^®^ 2 (Erbe Elektromedizin GmbH, Tübingen, Germany) system and 1.7, 1.9, or 2.4 mm cryoprobes, with smaller diameter cryoprobes used for tumors located in the upper lobes or more peripherally. Carbon dioxide (CO_2_) was used as the cryogenic gas. Afterwards, passive cryoprobe thawing for 60 s followed. The cooling–thawing stages were repeated for a total of 3 times to maximize tumor cell destruction in the treated area.

### 2.3. Assessment

Patients were followed up for up to 4 cycles of systemic therapy. Immune-related adverse events (irAEs) were evaluated using the National Cancer Institute Common Terminology Criteria for Adverse Events, version 5.0 (CTCAE v5.0) [20]. Grade 1 and 2 irAEs were attributed to immunotherapy by the treating physician or a consultant, specializing in the affected system, while grade 3 or higher irAEs were diagnosed only by the consultant.

Tumor imaging was performed using computed tomography (CT) during the time of the fourth cycle, which was 9 to 12 weeks from the beginning of systemic therapy. Tumor response was assessed according to RECIST 1.1 criteria [19]. Cases where a clear response could not be interpreted by radiologist were discussed in a multidisciplinary tumor board meeting. Patients who underwent bronchoscopic cryotherapy procedure also had a chest CT scan at the time of second cycle of systemic therapy. The three scans in cryotherapy group were evaluated for cavitation, necrosis, and surface area (defined as the product of its largest width and length on axial images).

### 2.4. Statistical Analysis

Due to the size of the study population, continuous variables such as patient age, treated tumor size, and target lesion changes are presented as medians with lower and upper quartiles. Categorical variables are described using frequencies and percentages for each group. The 95% confidence intervals (CI) for the overall response (ORR), described as the proportion of patients with complete (CR) or partial response (PR), and disease control rates (DCR), or the proportion of patients with CR, PR, or stable disease, were calculated using the Clopper–Pearson exact method. Differences in continuous variables between groups were assessed with the Mann–Whitney U test, while repeated measurements were analyzed using the Wilcoxon signed rank test. Categorical variables were evaluated using Fisher’s exact test and chi-square tests. Statistical analyses were conducted using SPSS version 29.0.1.0 (IBM Corp., Armonk, NY, USA). Exact tests were employed, with two-sided *p*-values below 0.05 considered statistically significant for population statistics and continuous variables, whereas one-sided *p*-values below 0.05 were deemed significant for overall response and disease control rates.

## 3. Results

**Figure 1:**
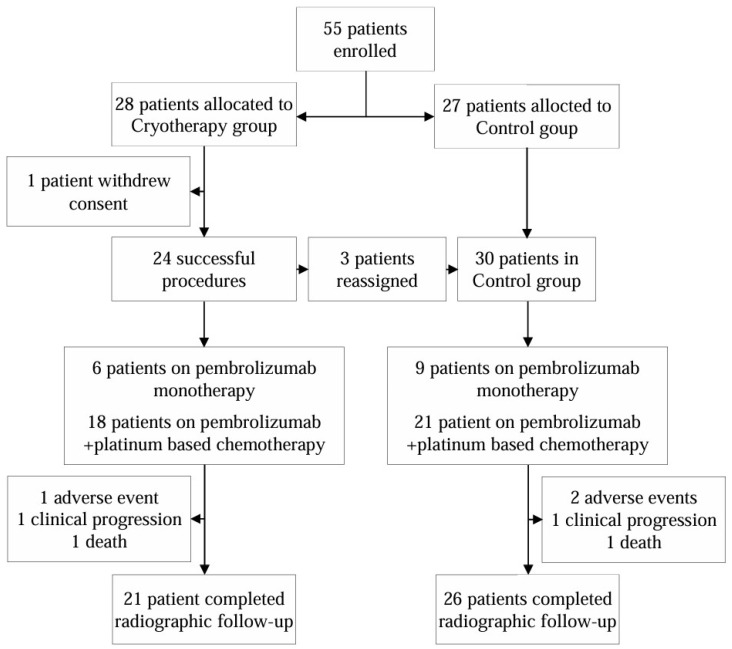
Enrollment and assignment to cryotherapy and control groups.

Between 27 February 2023 and 9 September 2024, a total of 55 patients were enrolled. Out of the 28 patients who were randomized to the cryotherapy group at first, 24 had a successful procedure, resulting in a technical success rate of 85.7%. Of all treated tumors, 11 (45.8%) tumors were treated via transbronchial cryotherapy and 13 (54.2%) with endobronchial cryotherapy. Of all patients treated via the transbronchial approach, only one had achieved the best cryoprobe location excentrically in relation to the tumor. Out of the four unsuccessful procedures, two failed due to inability to locate the pulmonary lesion via radial EBUS, and two failed due to the inability to position the cryoprobe within the tumor. As per protocol, these patients were offered to continue in the control group, with three agreeing and one patient withdrawing their consent to further participate in the study. The patient groups at the beginning and end of the study are provided in Figure 1.

The majority of patients were male (77.8%) and former or current smokers (88.9%). In total, 36 (66.7%) patients had at least one extrathoracic metastasis, with 16 (66.7%) patients in the cryotherapy group and 20 (66.7%) in the control group (*p* = 1.000). All patients received systemic treatment with pembrolizumab, although 39 (72.2%) patients received it with platinum-based chemotherapy. Carboplatin was the drug of choice for 34 (87.2%) of all the patients receiving chemotherapy—16 (88.9%) patients in the cryotherapy group and 18 (85.7%) in the control group (*p* = 1.000). Detailed population statistics are provided in Table 1.

Of the 24 patients in the cryotherapy group, 20 underwent CT scans both before and during the second and fourth cycles of systemic treatment. The excluded patients were those who did not complete the treatment course, as well as one patient who missed the second scan.

**Figure 2:**
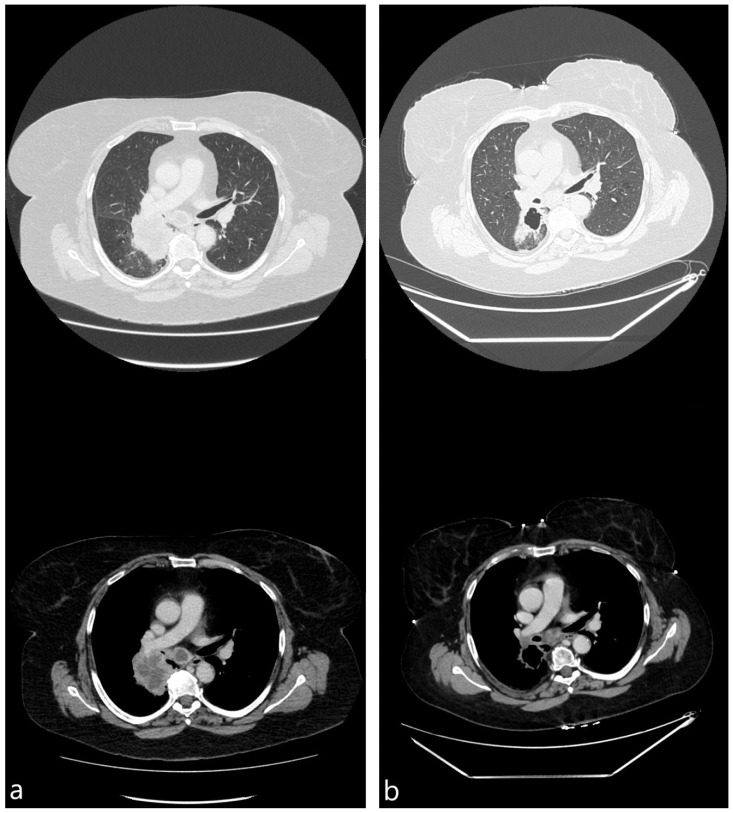
CT images, obtained 1 week before (**a**) and 4 weeks after (**b**) bronchoscopic cryotherapy (59 y.o. female with stage IV squamous cell carcinoma, PD-L1 expression 3%).

**Figure 3:**
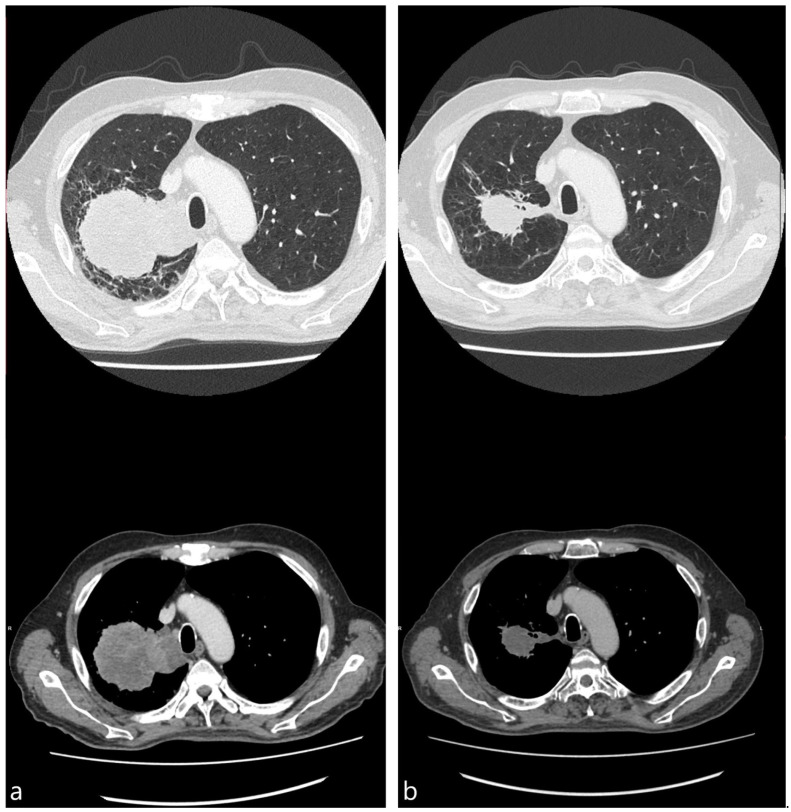
CT images, obtained almost 2 weeks before (**a**) and 4 weeks after (**b**) bronchoscopic cryotherapy (65 y.o. male with stage IV adenocarcinoma, PD-L1 expression 20%).

The median surface area of the treated tumors showed continuous shrinking from the baseline to the time of the fourth cycle, measuring 22.4 (10.1–62.3), 14.4 (10.6–26.6), and 10.2 (6.1–19.1) cm^2^ (*p* < 0.001). Additionally, 3 out of 20 (15.0%) patients had signs of cavitation in the tumor treated with cryotherapy at the baseline. This number increased to seven (35.0%) and eight (40.0%) during subsequent scans. Meanwhile, necrosis was observed in 12 (60.0%), 13 (65.0%), and 8 (40.0%) patients at the baseline, second, and fourth cycles, respectively. A pair of representative cases showing tumor reduction and cavitation are shown in Figure 2 and Figure 3.

As a pragmatic comparison, we also measured lung tumors in the control group with signs of being the primary tumor (e.g., single pulmonary tumor, signs of spiculation, etc.). The median surface area of these tumors decreased from 19.0 (7.8–34.9) to 10.0 (4.9–23.1) cm^2^ during treatment (*p* < 0.001). Cavitation was observed in four (15.4%) and six (23.1%) patients at the baseline and at the time of the fourth cycle, respectively. Meanwhile, necrosis was present in 9 (34.6%) and 11 (42.3%) cases.

Finally, we measured the surface area change of pulmonary tumors between the two groups. While the change appeared greater in the cryotherapy group, measuring at −42.9% (−16.4% to −73.6%), compared to −27.7% (−6.9% to −57.8%) in the control group, the difference was not statistically significant (*p* = 0.175).

Out of 47 patients who completed radiological follow-up, 12 (25.5%) showed a partial response after four cycles of systemic therapy. A total of 27 patients (57.5%) had stable disease. Meanwhile, eight patients had progressive disease (17.0%). The distribution of radiological outcomes in different study groups is provided in Table 2.

The ORR was 28.6% (95% CI, 11.3% to 52.2%) in the cryotherapy group and 23.1% (95% CI, 9.0% to 43.6%) in the control group (*p* = 0.461). Patients in the cryotherapy group had a relative risk of 0.81 (95% CI, 0.31 to 2.14) for no response compared to the control group. Meanwhile, patients in the cryotherapy group had a markedly better DCR. Only one patient in the cryotherapy group experienced progressive disease, resulting in a DCR of 95.2% (95% CI, 76.2% to 99.9%), compared to 73.1% (95% CI, 52.2% to 88.4%) in the control group (*p* = 0.049). The risk of disease progression in the cryotherapy group was 0.77 (95% CI, 0.60 to 0.99) times that of the control group.

**Figure 4:**
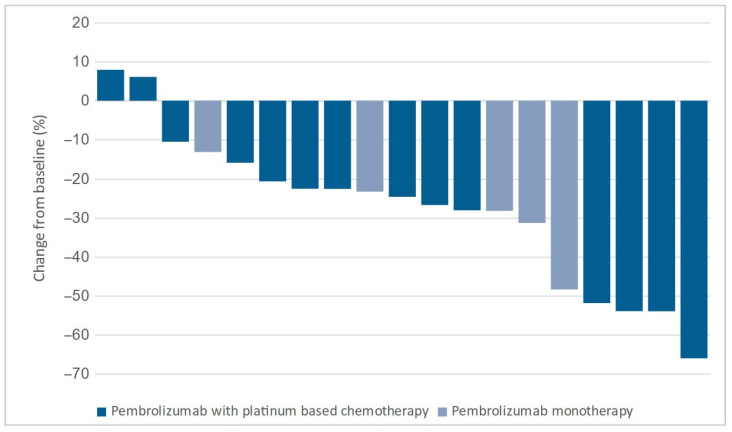
Changes in target lesion size in the cryotherapy group.

**Figure 5:**
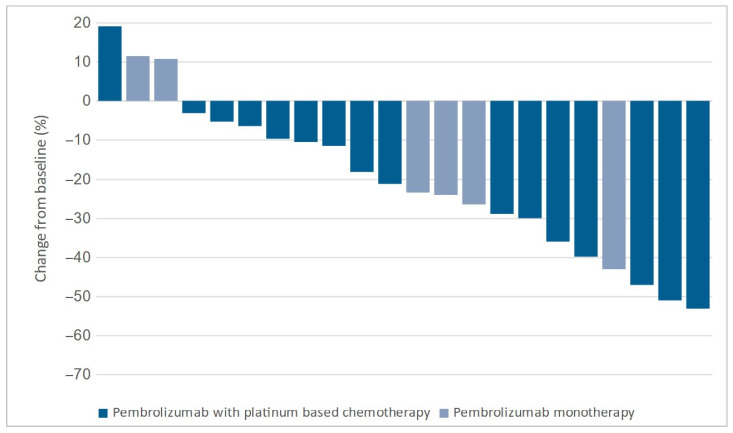
Changes in target lesion size in the control group.

No significant difference in the median change in target lesions between the groups was observed. The median change was −24.0% (−13.8% to −44.1%) in the cryotherapy group and −23.4% (−5.3% to −39.8%) in the control group (*p* = 0.296). Target lesion changes for both groups are shown in Figure 4 and Figure 5.

The period following the bronchoscopic therapy procedure was relatively uneventful. Two of the patients experienced subfebrile fever and one experienced mild hemoptysis, all successfully managed conservatively. One patient developed bacterial pneumonia, for which intravenous antibiotics had to be administered. No cases of serious bleeding during procedure or pneumothorax were observed.

The total number of patients who developed irAEs was 14 (25.9%), with 6 (25.0%) being in the cryotherapy group and 8 (26.7%) being in the control group (*p* = 1.000). One patient in the control group experienced two irAEs, those being hyperthyroidism and neuropathy. A total of three cases (5.6%) of grade 3 or higher irAEs were observed, with one in the cryotherapy group and two in the control group. The most frequent irAEs among both groups were hepatitis, hyperthyroidism, and nephritis, reported in four (7.4%), three (5.6%), and three (5.6%) patients, respectively. Despite premedication, there was a single grade 3 allergic reaction, which was found to be caused by paclitaxel. The distribution of irAEs between groups is provided in Table 3.

The median time to the first irAE did not differ between groups, being 45.5 (23–52.5) days in the cryotherapy group and 34 (25–45.5) days in the control group (*p* = 0.607). The single grade 3 irAE in the cryotherapy group occurred after 49 days, whereas the median time for the two patients in the control group was 31.5 days.

In total, seven patients, three from cryotherapy and four from the control group, discontinued systemic treatment due to adverse events, deteriorating ECOG performance status or, in the case of two patients, death.

## 4. Discussion

To our knowledge, this is one of the first studies examining the effects of cryotherapy and immunotherapy in metastatic NSCLC patients. The rationale for our research stems from the potential for these treatment modalities to overcome the shortfalls of one another.

Contact cryotherapy with a cryoprobe induces both immediate and delayed effects, resulting in tissue destruction. The immediate effect of cryotherapy is direct cell injury near the probe, caused by intracellular and extracellular ice crystal formation. The delayed effects arise from vasoconstriction and thrombosis, leading to tissue ischemia [9,21,22]. Meanwhile, cells located farther from the probe are not exposed to lethal temperatures and instead undergo apoptosis [21,22]. Necrosis results in the release of cancer cell neoantigens and damage-associated molecular patterns (DAMPs), which facilitate dendritic cell infiltration and maturation. Mature dendritic cells transport neoantigens to regional lymph nodes, presenting them to CD4 and CD8 T lymphocytes via major histocompatibility complex (MHC) class I and II molecules. The dendritic cells also co-present stimulatory proteins, such as CD80 and CD86 and secrete interleukins that are critical for Th1 immune responses, activating specific antitumor lymphocytes [23,24]. Conversely, the uptake of apoptotic cells does not lead to dendritic cell maturation. Instead, it induces a tolerogenic state in dendritic cells, resulting in T cell anergy [22,25]. As necrosis promotes immune activation and apoptosis-immune tolerance, the systemic effects of cryotherapy arise from the balance between these two processes.

An activated immune system has intrinsic mechanisms, such as immune checkpoints, that are in place to prevent prolonged inflammation and minimize damage to healthy tissues. Cancer cells exploit these checkpoints as escape mechanisms, blocking further T cell proliferation and suppressing the continuation of the antitumor immune response. Several studies using murine models have reported increased numbers of PD-1-expressing T cells and the upregulation of PD-L1 expression on tumor cells following tumor cryoablation [12,16,26]. The addition of PD-1/PD-L1 axis inhibitors enables T cells to sustain the antitumor response, effectively destroying both residual tumor tissue after cryoablation and distant metastases [12,16].

In our study, both the pulmonary lesions treated with cryotherapy and those in the control group showed a significant decrease in surface area. This finding suggests that the antitumor immune response, rather than direct cell destruction by cryotherapy, was the primary driving factor behind this effect, and cryotherapy had an insignificant additional effect against established tumors. Conversely, tissue destruction in the cryotherapy group might have been offset by increased infiltration of immune cells. While we did not perform repeat biopsies after cryotherapy to evaluate this possibility, a recent study of eight metastatic NSCLC patients by Desilets et al. found significant upregulation of tumor-infiltrating CD8 T cells in cases where a clinical benefit from immunotherapy with pembrolizumab was observed [27].

Interestingly, our study observed an apparent increase in cavitation in tumors treated with cryotherapy. Cavitation in treatment-naive NSCLC has been associated with a worse prognosis by some authors [28,29]. While Nguyen et al. did not report a similar association with survival, they noted that cavitating NSCLCs were associated with a larger size and greater metabolism on PET/CT scans [30]. Interestingly, Chaudhry et al. found that patients who underwent percutaneous cryoablation for primary stage I lung cancer and developed cavitation did not experience disease recurrence [31]. Cavitation in treatment-naive tumors appears to indicate more aggressive cancer growth, whereas in treated tumors, it seems to represent treatment success and is associated with better outcomes.

While we observed no significant differences between the studied groups in terms of objective response, the cryotherapy group demonstrated increased resistance to disease progression. A robust molecular basis underpins these findings. The potential of cryotherapy to induce a systemic immune response and prevent metastasis was demonstrated by Sabel et al. in a study using 4T1 mammary carcinoma-bearing Balb/c mice [32]. In their study, primary tumors were treated with cryoablation using either a low or high freeze rate, removed surgically, or left untreated. High freeze rate cryoablation resulted in the lowest count of pulmonary metastases (4.89 nodules per mouse), compared to surgical excision (9 nodules per mouse) and untreated controls (47.2 nodules per mouse). Subsequently, lymphocytes from tumor-draining lymph nodes were extracted and cultured with either 4T1 or RENCA tumor cells as a control. Lymphocytes from mice treated with high-freeze-rate cryoablation exhibited increased IFN-γ production after co-culture with 4T1 cells but not RENCA cells, suggesting a tumor-specific immune response. Notably, low freeze rates did not produce a similar effect.

However, the transience of the antitumor response elicited by cryotherapy alone was highlighted by Zhu et al. [12]. In their study, mice inoculated with RENCA cells on bilateral flanks received cryoablation on one side. Examination of the contralateral tumor revealed upregulation of IFN-γ and GZMB on day 3, followed by a decline by day 7. Concurrently, PD-L1 mRNA expression increased. Combining cryoablation with anti-PD-1 therapy significantly decreased distant tumor growth compared to either treatment alone. Similar results were reported by Liu et al. in a murine Lewis lung adenocarcinoma model [13]. In addition to reduced abscopal tumor growth, the combination of cryoablation and anti-PD-1 therapy induced immune memory, resulting in resistance to rechallenge with Lewis lung carcinoma cells but not MC38 colon cancer cells.

The translation of combined cryotherapy and immunotherapy’s effectiveness seen in murine models to a clinical setting is mainly limited to several case reports and small studies [14,15,16,27]. Additionally, one retrospective study examined combined effect of argon–helium cryoablation and monoclonal anti-PD-1 antibody nivolumab immunotherapy in patients with stage IIIb-IV NSCLC, who had a relapse after radiotherapy, surgical resection or radical radiotherapy, or disease progression after chemotherapy [17]. While the comparator was cryoablation alone and not immunotherapy, that study found similar benefits in DCR, but not ORR, as reported by our study. It is possible, that cryotherapy and immunotherapy could synergize to prevent cancer cells from forming metastases, although further research is required to confirm this.

Additional large controlled studies investigating the efficacy of cryotherapy in NSCLC treatment are currently underway (NCT03290677, NCT04049474, NCT04339218, NCT04793815, NCT06000358). Meanwhile, other treatment modalities have also been examined for synergy with immunotherapy. The PEMBRO-RT (NCT02492568) and MDACC (NCT02444741) trials examined the safety and effectiveness of pembrolizumab, with or without radiotherapy. A pooled analysis of these two studies revealed an abscopal response rate of 41.7% in the pembrolizumab-and-radiotherapy group, compared to 19.7% with pembrolizumab monotherapy, as well as increased progression-free and overall survival [33].

We observed no significant complications during bronchoscopic cryotherapy procedures. Bronchoscopy offers a relatively safe and efficient way for cryotherapy for lung tumors and is frequently used for the treatment of endobronchial lesions. Various studies have identified bleeding and pneumothorax as the primary complications of endobronchial cryotherapy, with reported rates of 0.7–12% and 0.1–0.7%, respectively [8]. The main limitations of current bronchoscopic cryotherapy include the difficult positioning of the cryoprobe to fully encompass the lesion, especially compared to percutaneous techniques, and the lower cooling capability of flexible cryoprobes compared to rigid ones [34]. The development of robotic bronchoscopy, advanced imaging techniques, such as cone beam CT, and improved cryoprobe designs may help mitigate these disadvantages in the future.

The safety profile concerning irAEs observed in our study was mainly consistent with previous studies of pembrolizumab treatment for metastatic NSCLC [5,6]. There was no increase in the total number of irAEs after cryotherapy, compared to controls. Two of the most observed irAEs in our study—hyperthyroidism and hepatitis—have also been reported to be among the earliest to arise, with the median time to onset reported at 43 and 63 days, respectively [35].

## 5. Limitations

Our study has several limitations, primarily a small sample size and a short follow-up period, which may have impacted the significance of some findings. This also prevented a subgroup analysis regarding such important factors as PD-L1 expression, the type of systemic treatment, or histological cancer type. Additionally, the lack of completed clinical studies involving metastatic NSCLC patients treated with a combination of cryotherapy and immunotherapy precludes direct comparisons. Despite these limitations, we believe our findings provide reasonable evidence to justify further research in this field.

## 6. Conclusions

Our study demonstrated that combined bronchoscopic cryotherapy and immunotherapy with or without chemotherapy may decrease the rate of progressive disease in metastatic non-small cell lung cancer, while maintaining a satisfactory safety profile.

## Figures and Tables

**Table 1:** Demographic, disease, and treatment characteristics of patients.

	Cryotherapy (*n* = 24)	Control (*n* = 30)	Exact Significance
Median age—years (range)	64 (62–71)	64 (60–72)	0.841
Male—*n* (%)	19 (79.2)	23 (76.7)	1.000
Smokers—*n* (%)	21 (87.5)	27 (90.0)	1.000
Performance status—*n* (%)			
0	5 (20.8)	7 (23.3)	1.000
1	19 (79.2)	23 (76.7)	
Histology—*n* (%)			
Squamous	11 (45.8)	13 (43.3)	1.000
Adenocarcinoma	13 (54.2)	17 (56.7)	
PD-L1 tumor proportion score—*n* (%)			
<1%	11 (45.8)	12 (40.0)	0.940
1–49%	7 (29.2)	9 (30.0)	
≥50%	6 (25.0)	9 (30.0)	
Brain metastases—*n* (%)	3 (12.5)	5 (16.7)	0.720
Pembrolizumab monotherapy—*n* (%)	6 (25.0)	9 (30.0)	0.766

*n*—number of patients; PD-L1—programmed death-ligand 1.

**Table 2:** Comparison of radiological response between study groups.

	Partial Response	Stable Disease	Progressive Disease
Cryotherapy—*n* (%)	6 (28.6)	14 (66.7)	1 (4.7)
Control—*n* (%)	6 (23.1)	13 (50.0)	7 (26.9)
Total—*n* (%)	12 (25.5)	27 (57.5)	8 (17.0)

*n*—number of patients.

**Table 3:** Immune-related adverse events.

	Cryotherapy (*n* = 24)		Control (*n* = 30)		Total (*n* = 54)	
Immune-related adverse events	Any grade	Grade ≥ 3	Any grade	Grade ≥ 3	Any grade	Grade ≥ 3
Number of patients (%)						
Any	6 (25.0)	1 (4.2)	9 (30.0)	2 (6.7)	15 (27.3)	3 (5.5)
Hepatitis	2 (8.3)	1 (4.2)	2 (6.7)	1 (3.3)	4 (7.3)	2 (3.6)
Hyperthyroidism	2 (8.3)	0 (0.0)	1 (3.3)	1 (3.3)	3 (5.5)	1 (1.8)
Hypophysitis	1 (4.2)	0 (0.0)	0 (0.0)	0 (0.0)	1 (1.8)	0 (0.0)
Nephritis	1 (4.2)	0 (0.0)	2 (6.7)	0 (0.0)	3 (5.5)	0 (0.0)
Colitis	0 (0.0)	0 (0.0)	2 (6.7)	0 (0.0)	2 (3.6)	0 (0.0)
Hyperparathyroidism	0 (0.0)	0 (0.0)	1 (3.3)	0 (0.0)	1 (1.8)	0 (0.0)
Neuropathy	0 (0.0)	0 (0.0)	1 (3.3)	0 (0.0)	1 (1.8)	0 (0.0)

## Data Availability

The data presented in this study are available on request from the corresponding author.
